# Evaluation of Growth Hormone Therapy in Seven Chinese Children With Familial Short Stature Caused by Novel *ACAN* Variants

**DOI:** 10.3389/fped.2022.819074

**Published:** 2022-03-07

**Authors:** Jie Sun, Lihong Jiang, Geli Liu, Chen Ma, Jiaqi Zheng, Lele Niu

**Affiliations:** Department of Pediatrics, Tianjin Medical University General Hospital, Tianjin, China

**Keywords:** familial short stature, *ACAN* gene, novel variants, prevalence, recombinant human growth hormone

## Abstract

**Objective:**

*ACAN* gene variants are an important cause of familial short stature (FSS). Appropriate growth-promoting therapies effectively improve the patient height. Here, we report a therapeutic assessment of cases of seven families of FSS patients with heterozygous *ACAN* variants. Our findings provide a valuable theoretical basis for the clinical diagnosis and treatment of this disease.

**Methods:**

From December 2020 to June 2021, 32 FSS patients were examined in Tianjin Medical University General Hospital (Tianjin, China) by whole-exome sequencing to determine whether *ACAN* variants were present. Their clinical data were summarized and scrupulously analyzed.

**Results:**

We found seven novel heterozygous *ACAN* variants: c.1051 + 2T > A, c.313T > C (p.S105P), c.2660C > G (p.S887X), c.2153C > A (p. T718K), c.7243delG (p.D2415Tfs^*^4), c.2911G > T (p.G971X), c.758-7T > C. All seven patients had proportionate short stature and mild skeletal dysplasia. Endocrine examination results were normal. Only one of the patients had an advanced bone age (1.1 years older than chronological age), whereas the other patients had normal bone ages. All of them had a family history of short stature, with or without osteoarthritis or intervertebral disc disease. All seven patients accepted treatment with recombinant human growth hormone (rhGH) and were regularly followed up. One patient did not come at the follow-up visit. The height of the remaining six patients before and after the treatment was −2.89 ± 0.68 SDS, −1.91 ± 0.93 SDS, respectively, with a treatment course of 1.85 ± 1.91 years. A good therapeutic response was observed in all of them.

**Conclusions:**

In this study, seven novel heterozygous variants in *ACAN* were discovered, which expanded the spectrum of the already established *ACAN* pathogenic variants. In FSS cohort, the proportion of *ACAN* variants accounted was large. The treatment with rhGH effectively increased the patient height, but further studies with longer follow-up periods and more extensive observations are required to elucidate the long-term effect.

## Introduction

The linear growth of bones is determined by proliferation and differentiation of the growth plate chondrocytes. Endocrine and paracrine factors, extracellular matrix molecules, and intracellular proteins jointly regulate the formation of the growth plate cartilage and participate in the maintenance of its normal function (1). Genetic defects affecting the growth plate development and functions could cause short stature. Encoded by the *ACAN* gene, aggrecan is a major proteoglycan that is an important component of the cartilage extracellular matrix, with a key role in cartilage and bone morphogenesis. Pathogenic *ACAN* variants could result in a wide spectrum of clinical phenotypes. For example, homozygous or compound heterozygous variants of the *ACAN* gene could cause spondyloepimetaphyseal dysplasia (SEMD); on the other hand, heterozygous variants could induce spondyloepiphyseal dysplasia Kimberley type (SEDK), familial osteochondritis dissecans, and idiopathic short stature (ISS), with or without mild skeletal dysplasia and advanced bone age (2, 3). The clinical phenotypes of patients are quite different, but significant short stature and familial short stature (FSS) are among the most commonly occurring. In this study, the clinical data of seven FSS families with *ACAN* variants were summarized and the results were analyzed, which provides a theoretical basis for the clinical diagnosis and treatment of this disease.

## Materials and Methods

### Patients and Study Design

From December 2020 to June 2021, 32 FSS patients with 19 males (59.4%) and 13 females (40.6%) in Tianjin Medical University General Hospital (Tianjin, China) underwent whole-exome sequencing. Based on the results, heterozygous variants in *ACAN* were identified in seven of them. FSS usually 2 indicates that the patient height and those of the parents (or one of them) are less than the average height by two height standard deviations, of the population of the same age, gender, and race. The height of patients was assessed using the “Height and weight standards for Chinese children and adolescents aged 0–18 years.”

All seven patients were advised to receive recombinant human growth hormone (rhGH) therapy with a dosage of 0.15 IU·(kg·d)^−1^ firstly. Furthermore, patients were suggested to perform regular visits every 3 months for monitoring their physical development conditions, including the changes in their heights and weights, and to undergo laboratory examinations, including blood routine tests, as well as tests of thyroid function, insulin-like growth factor 1 (IGF1), insulin-like growth factor-binding protein 3, 25-hydroxy vitamin D3 (25-OHD3), fasting blood glucose and insulin. Other examinations were performed once every 6 months, including liver and kidney function, blood electrolytes, bone age and spinal radiological assessment. Pituitary MRI was also conducted once a year. To evaluate the therapeutic efficacy, the change in the height standard deviation score (SDS) was calculated, and a height growth curve was drawn. The dosage of rhGH was adjusted based on the height growth and laboratory examination results. The oral vitamin D dosage was adjusted according to the 25-OHD3 test results obtained at each follow-up examination. Specifically, 800 IU/d was given if the 25-OHD3 was <50 nmol·L^−1^, and 400 IU/d was given if its level was 50–75 nmol·L^−1^.

The clinical data of these seven patients were collected, including their clinical manifestations, family history, physical examination, biochemical, and radiological examination results, and treatment status. This study was approved by the Ethics Committee of Tianjin Medical University General Hospital. The parents of the patients signed informed consent forms before study initiation.

### Genetic Analysis

Approximately 2 ml of peripheral blood were drawn from each family member in tubes containing EDTA as anticoagulant. To make a definite diagnosis, we performed whole-exome sequencing (WES) of the proband of each family included. We fragmented 1–3 μg of genomic DNA, extracted from each sample, to an average size of 180 base pairs (bp) using a Bioruptor sonicator (Diagenode, Liège, Belgium). Paired-end sequencing libraries were then prepared using a DNA sample-prep reagent set 1 (New England Biolabs UK Ltd., Hitchin, UK). Library preparations, including end repair, adapter ligation, and PCR enrichment, were performed following Illumina protocols. Amplified DNA was captured using GenCap Short-statue Capture Kit (MyGenostics GenCap Enrichment Technologies, Chongqing, China). The capture experiment was conducted in accordance with the manufacturer's protocol. Then, the PCR product was purified using SPRI beads (Beckman Coulter, Brea, CA, USA) following manufacturer's instructions. The enrichment libraries were next sequenced using an Illumina Nova6000 sequencer (Illumina, San Diego, CA, USA) for paired reads of 150 bp. After sequencing, the raw data were saved in a FASTQ format and analyzed. Illumina sequencing adapters and low-quality reads (<80 bp) were filtered according to the Cutadapt quality-controlled process (version 1.16, https://cutadapt.readthedocs.io/en/stable/guide.html). After quality control, the clean reads were mapped to the UCSC hg19 human reference genome using Burrows-Wheeler Aligner (version 0.7.12-r1044) software. Duplicated reads were removed using Picard tools (version 2.2.3) and mapped reads were utilized for variation detection. Furthermore, variants were annotated by ANNOVAR software and associated with multiple databases, such as the 1,000 Genomes dataset, ESP6500, NCBI dbSNP, EXAC, HGMD. Prediction was then implemented by software, including SIFT, PolyPhen-2, Mutation Taster, GERP++. The candidate variants were validated by Sanger sequencing, and their pathogenicity was graded based on the American College of Medical Genetics and Genomics (ACMG) guidelines (4).

## Results

### Clinical Features

**Figure 1:**
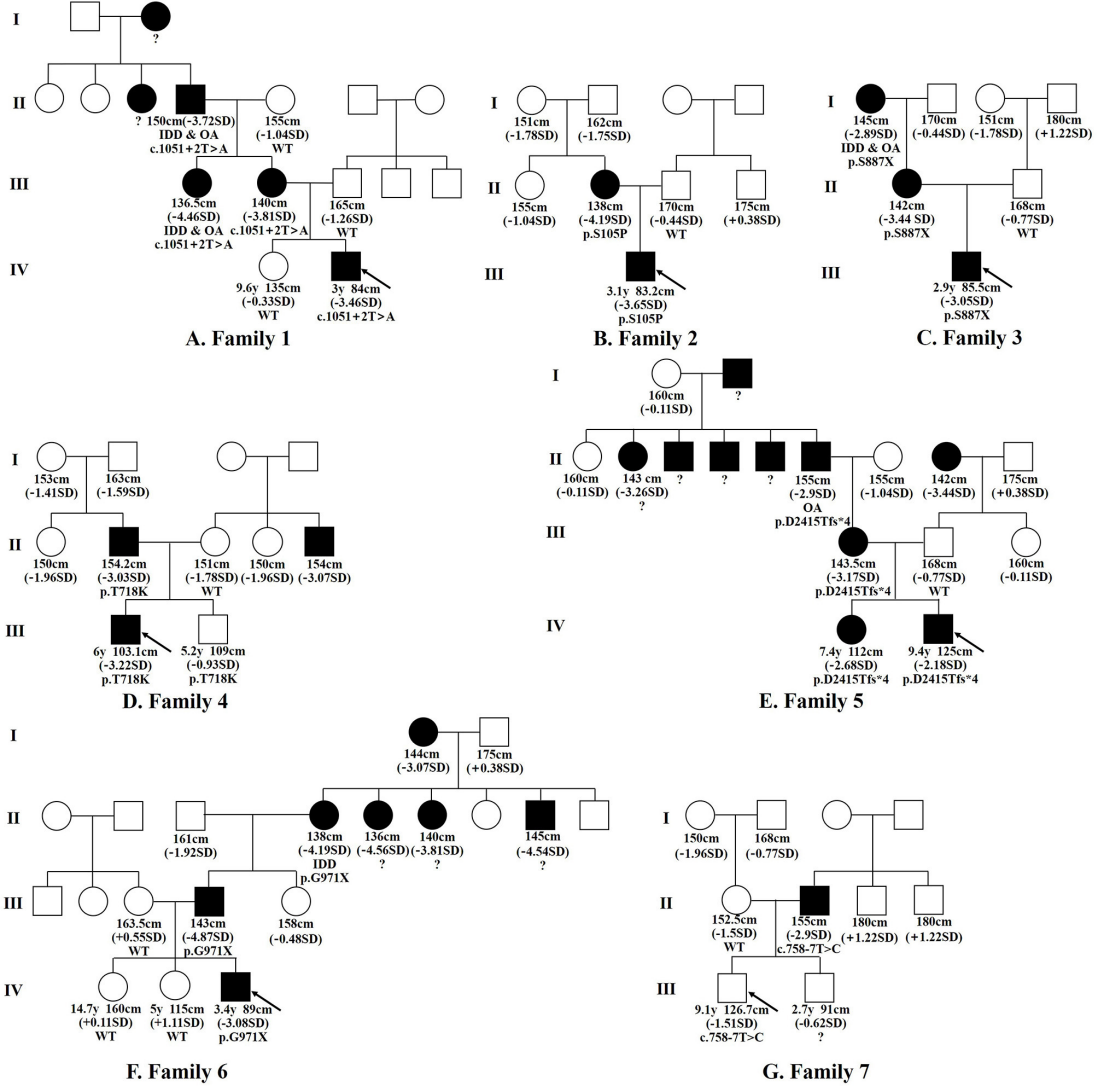
Family tree diagram. Pedigree analysis of the *ACAN* variations in the seven families examined. Black icons represent the individuals with short stature (< -2SD). The black arrow indicates the proband. y, years; SD, standard deviation; OA, osteoarthritis; IDD, intervertebral disc disease; ?, unknown genotype or phenotype.

The seven FSS patients studied were boys. The range of age at first diagnosis was from 2.9 to 9.4 years. The range of birth length was 48–50 cm (−1.35 SDS to −0.22 SDS), and the range of birth weight was 2.44–3.30 kg (−2.44 SDS to −0.05 SDS). Only BMI-SDS of patient 3 was < -2, which was in line with the diagnosis of patient with small-for-gestational-age (SGA). The height SDS was −2.88 ± 0.71 (−3.65 SDS to −1.51 SDS), and all patients had different degrees of short stature. The weight SDS of the patients was −2.11 ± 0.77 (−3.45 SDS to +0.51 SDS). The ratio of the sitting height to the lower limb length was in the range of P3–P97, suggesting proportionate short stature. The major skeletal deformities were frontal bossing, flat nasal bridge, mild midface hypoplasia, short neck, spinal deformity, short fingers, short thumbs, broad great toes, and the second toe longer than other toes. None complained of bone or joint pain. Intelligence and motor development were normal. The seven patients had a family history of short stature, with the height SDS of the 12 affected adults of −3.63 ± 0.65 (−2.89 SDS to −4.87 SDS). Four patients had a family history of intervertebral disc disease or osteoarthritis (patients 1, 3, 5, and 6), as can be observed in the family tree illustrated in Figure 1. The skeletal deformities of the seven affected adults included mild facial deformities, short necks, arm span longer than height, limited elbow extension, knee varus, wide fingers, short thumbs, broad great toes, and the second or third toe longer than the other toes.

**Figure 2:**
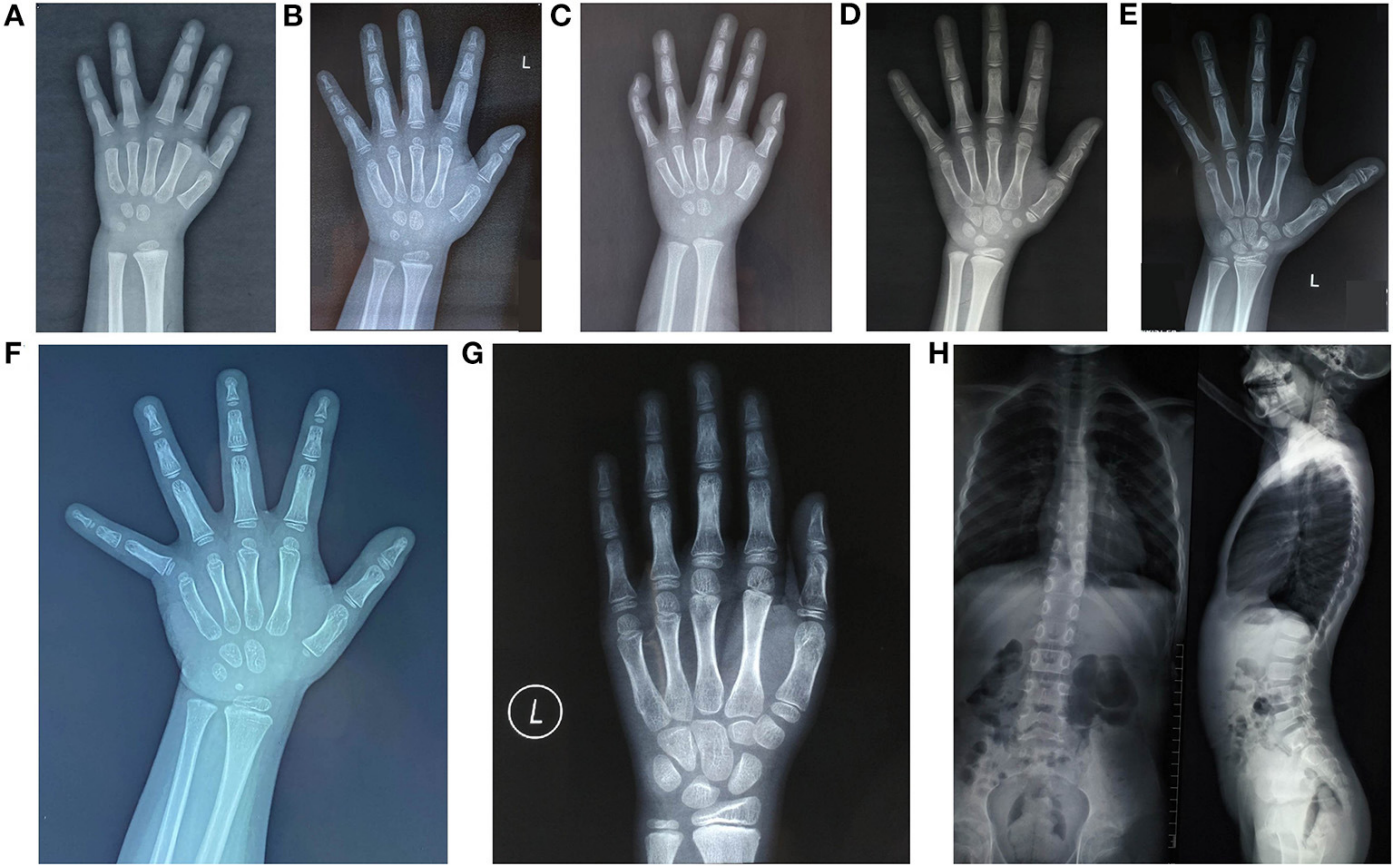
Radiology imaging data of the seven patients studied. **(A)** Patient 1, BA 2.6 years old, CA, 3 years old; **(B)** Patient 2, BA, 3.6 years old, CA, 3.1 years old, with a short thumb; **(C)** Patient 3, BA, 2 years old, CA, 2.9 years old, the 5th distal phalanx bended inward; **(D)** Patient 4, BA, 8.2 years old, CA, 7.3 years old; **(E)** Patient 5, BA, 10 years old, CA, 9.4 years old, with a short thumb; **(F)** Patient 6, BA, 3.8 years old, CA 3.4 years old, with a short thumb; **(G, H)** Patient 7, the left-hand X-ray showed BA, 10.2 years old and CA, 9.1 years old; spine lateral X-ray revealed mild thoracolumbar scoliosis. P, proband; BA, bone age; CA, age.

The laboratory examinations showed no obvious abnormalities in the calcium and phosphorus metabolism, and all endocrine tests for short stature were normal. The initial value of IGF1 was 0.05 ± 0.57 SDS (−0.9 SDS to 0.8 SDS). Compared with chronological age, six patients had a normal bone age (<1 year younger or older than chronological age) and normal spine. One patient had an advanced bone age by 1.1 years, with mild thoracolumbar scoliosis (patient 7), as can be seen in Figure 2.

**Figure 3:**
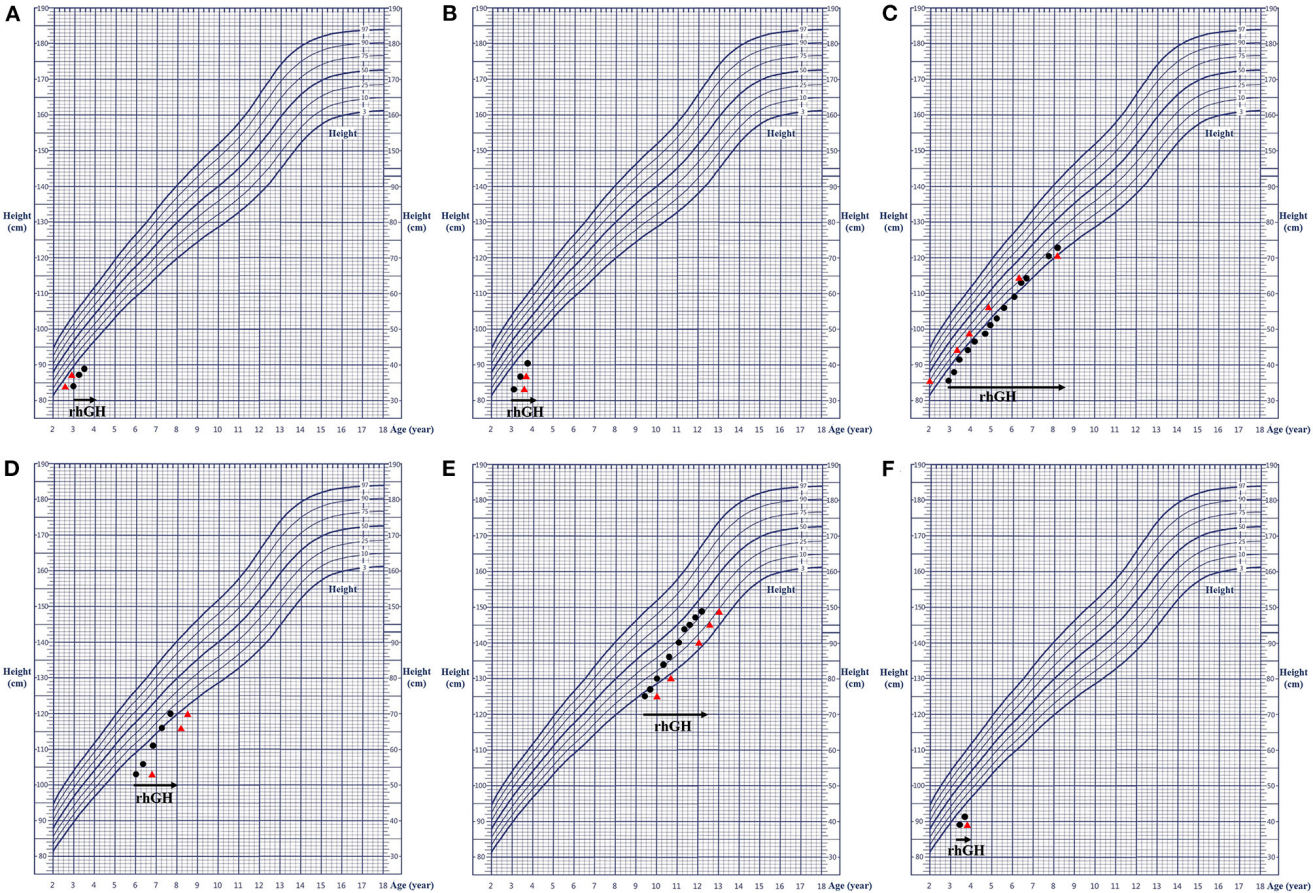
Growth charts of the six patients. **(A–F)** corresponding to patients 1–6, respectively. The black dot represents the height at the respective age; the red triangle indicates the height at the corresponding bone age; and the black arrow shows the rhGH treatment. rhGH, recombinant human growth hormone.

All these seven patients received rhGH treatment, of which 1 case (patient 7) did not come at the follow-up examination visit. The treatment course of the remaining six patients lasted 1.85 ± 1.91 years, with a rhGH dosage applied during the treatment with 0.155 ± 0.016 IU·(kg·d)^−1^. The height of them before and after treatment was −2.89 ± 0.68 SDS and −1.91 ± 0.93 SDS, respectively, indicating a good response to the treatment. The height growth curve was presented in Figure 3. No adverse reactions were observed, and all laboratory and radiological examinations had normal results in all patients during the treatment. The IGF1 of six patients was 1.19 ± 0.64 SDS (−0.4 SDS to 2.5 SDS). Furthermore, no scoliosis and no obvious acceleration of the bone age were detected in the patients. The detailed follow-up information of the six patients is presented in Supplementary Table 1, and the detailed clinical characteristics of the seven patients are listed in Table 1.

**Table 1:** Clinical data of the seven patients included in the study.

	**Patient number**	**P1**	**P2**	**P3**	**P4**	**P5**	**P6**	**P7**
Basic information	**Gender**	**M**	**M**	**M**	**M**	**M**	**M**	**M**
	Age of first Visit (y)	3	3.1	2.9	6	9.4	3.4	9.1
	Height (cm)	84	83.2	85.5	103.1	125	89	126.7
	Height SDS	−3.46	−3.65	−3.05	−3.22	−2.18	−3.08	−1.51
	Weight (kg)	10	11.5	11	17.5	35.7	13.5	24
	Weight SDS	−3.45	−2.25	−2.67	−1.56	0.51	−1.35	−1.39
	BMI-SDS	−0.28	1.15	−2.36	−0.18	0.09	−0.69	−0.28
Birth and growth history	Gestational age (w)	40	37	38^+4^	40^+6^	40^+4^	39	39^+6^
	BL (cm)	50	48	48	49	50	49	50
	BW (kg)	3.2	3.3	2.44	3.1	3.3	2.97	3.2
	Intelligence	N	N	N	N	N	N	N
	Motor development	N	N	N	N	N	N	N
Family history	Short stature	+	+	+	+	+	+	+
	Intervertebral disc disease	+	-	+	-	-	+	-
	Osteoarthritis	+	-	-	-	+	-	-
Special symptoms and signs	Head circumference (cm)	47.8	48.3	48	NA	NA	49	51.5
	Sitting height (cm)	51	48.8	49.8	57.3	65.5	51	69
	Arm span (cm)	83.5	81.3	86.2	101.5	128	89.1	124.5
	Midface hypoplasia	+	-	-	-	-	-	-
	Frontal bossing	+	+	-	-	-	+	-
	Flat nasal bridge	+	+	-	-	-	+	-
	Long philtrum	-	+	+	-	-	-	-
	Short neck	+	+	+	-	+	+	-
	Osteochondritis/osteoarthritis	-	-	-	-	-	-	-
	Brachydactyly	+	+	-	+	-	+	+
	Short thumbs	-	+	-	-	+	+	-
	Broad great toes	-	-	+	+	-	-	-
	Long second toes	+	-	+	-	-	-	-
Laboratory test	Blood routine	N	N	N	N	N	N	N
	Liver and kidney function	N	N	N	N	N	N	N
	Blood electrolytes	N	N	N	N	N	N	N
	Thyroid function	N	N	N	N	N	N	N
	Fast blood glucose and insulin	N	N	N	N	N	N	N
	GH (ng/ml)	NA	0.75	4.2	1.6	0.1	0.44	4.18
	GH peak (ng/ml)	NA	NA	NA	7.39	2.33	NA	NA
	IGF-1 (ng/ml)	37	56	60	151	205	91	234
	IGF-1 SDS	−0.88	−0.12	0.02	0.42	0.09	0.78	0.3
	IGFBP3 (ug/ml)	2.81	3.55	2.51	4.54	5.28	3.44	4.38
	25-OHD3 (nmol/L)	27.1	85.28	60.65	40.72	33.14	112.59	35.43
Radiological examination	Bone age (y)	2.6	3.6	2	6.8	10	3.8	10.2
	BA-CA	−0.4	0.5	−0.9	0.8	0.6	0.4	1.1
	Scoliosis	-	-	-	-	-	-	+
	Pituitary MRI	N	N	N	N	N	N	N
rhGH treatment	Start age (y)	3	3.1	2.9	6	9.4	3.4	9.3
	Height SDS	−3.46	−3.65	−3.05	−3.22	−2.18	−3.08	−1.61
	Treatment duration (years)	0.5	0.67	5.25	1.67	2.75	0.25	—
	Height SDS after treatment	−3.13	−2.68	−1.35	−1.37	−0.44	−2.46	—
	Change in height SDS	0.33	0.97	1.7	1.85	1.74	0.62	—

*P, patient; M, male; y, years old; SDS, standard deviation score; BL, birth length; BW, birth weight; BMI, body mass index; GH, growth hormone; GH peak, the peak of the growth hormone during the growth hormone stimulation test; IGF1, insulin-like growth factor 1; IGFBP3, insulin-like growth factor-binding protein 3; 25-OHD3, 25-hydroxy vitamin D3; rhGH, recombinant human growth hormone; NA, not available; ‘+' and ‘-' indicate “yes” and “no”, respectively*.

### Genetic Discovery

We conducted whole-exome sequencing in a cohort of 32 FSS patients and found that seven of them carried *ACAN* heterozygous variants, with a detection rate of 21.9% (7/32). The *ACAN* variants in patients 1–7 were c.1051+2T > A, c.313T > C (p.S105P), c.2660C > G (p.S887X), c.2153C > A (p.T718K), c.7243delG (p.D2415Tfs^*^4), and c.2911G > T (p.G971X), c.758-7T > C, respectively. All seven variants were novel, including two splicing, two missense, and two non-sense variants, as well as one frame shift variant. The genetic information is displayed in Supplementary Table 2. Sanger sequencing showed that the *ACAN* variants of the seven patients were inherited from affected parents (Figure 1), with a dominant inheritance pattern. Among the 21 affected individuals with *ACAN* variants from seven families including 13 males (61.9%) and 8 females (38.1%) (Figure 1), 19 individuals were with short stature (19/21), indicating a remarkably high penetrance rate.

## Discussion

The *ACAN* gene is located on chromosome 15q26.1 and contains 19 exons. Exons 2-19 are protein coding, including a 2530 amino acid isoform. Aggrecan regulates the expression of key growth factors and signal molecules during the development of the cartilage tissue and is thus essential for the structure, morphology, and survival of the cartilage cells (5). Notably, heterozygous *ACAN* variants were detected in 1.4–4.6% of the ISS patients or from 2.5 to 6% of the FSS patients in small cohort studies in patients with different ethnic backgrounds (3, 6–8). Recently, a large Chinese ISS cohort study found that the proportion of *ACAN* gene with pathogenic variants in the entire cohort was 1.2% (12/1005), whereas its proportion in the FSS sub-cohort was 3.5% (8/229) (9). However, in our investigation, the proportion of the *ACAN* variants in the FSS patients was significantly higher (21.9%, 7/32), which might have been associated with the single location and small sample size. Although the proportions established in these aforementioned studies were different, they all indicated that the *ACAN* gene variant was a significantly important cause of ISS or FSS. Although most of the patients were born with a normal birth length and weight, a certain percentage of SGA patients were also detected. Freire et al. (10) reported that heterozygous *ACAN* variants accounted for 1.8% (1/55) of the SGA patients and 12.5% (1/8) of the SGA patients carrying genetic variations. Recently, Liang et al. (11) summarized and assessed the clinical data of 64 patients with *ACAN* heterozygous variants and found a SGA prevalence of 15.5% (10/64). In our study, we detected seven novel heterozygous variants of *ACAN*. Patients 1 and 2 and patients 4–7 were initially diagnosed with ISS, whereas patient 3 was initially diagnosed with SGA. All these patients had proportionate short stature, slight skeletal dysplasia, and normal endocrine examination results. In summary, *ACAN* variants were not only related to ISS and FSS, but might have also been associated with SGA. Therefore, *ACAN g*ene screening in these patients is highly necessary.

Patients with heterozygous *ACAN* variants are mainly manifested as proportionate or slightly disproportionate short stature, with potentially advanced, normal, or delayed bone age. They may also be accompanied by non-specific skeletal abnormalities, such as frontal bossing, mild midface dysplasia, flat nasal bridge, short neck, short fingers, short thumbs, short metacarpal bones, broad great toes, lumbar lordosis, and scoliosis (11, 12). Four of these seven patients finally included in our study had mild facial deformities (such as frontal bossing, mild midface dysplasia, flat nasal bridge, and long humans, 4/7). Five patients had short necks (5/7). Five patients had short fingers (5/7). Three had short thumbs (3/7), and one had abnormal palm prints (1/7). Moreover, two of the patients had broad great toes (2/7), two with second toe longer than other toes (2 /7), and one with scoliosis (1/7). In addition, studies have shown that some patients were accompanied by early-onset osteoarthritis, osteochondritis and intervertebral disc disease. In an international cohort investigation of 103 patients from 20 families (12), 12 families were found to have developed early-onset osteoarthritis that had started in late puberty and whose most common manifestation was knee pain. Three families had also osteochondritis dissecans, and 11 families had degenerative disc disease, which had begun between the fourth and fifth decades of life. Liang et al. (11) found that approximately one-third of the heterozygous *ACAN* variant carriers developed osteochondritis or osteoarthritis. Thus, these manifestations are not common, but they may develop with age. The patients may have an incomplete phenotype before puberty. In this study, seven patients were very young at first visit and only one patient entered puberty during the follow-up. None of the patients developed bone diseases. However, of the 12 adult individuals with *ACAN* variants, 5 cases developed osteoarthritis or intervertebral disc diseases. Therefore, the future follow-up of these seven patients in this study should pay attention to not only the height, but also bone diseases. Nilsson et al. (13) firstly reported that *ACAN* heterozygous variants could lead to advanced bone age and early growth arrest. Subsequent investigations in subjects of different ethnic backgrounds have also revealed that *ACAN* variants were associated with short stature with advanced bone age (12, 14–16). However, recent studies (3, 7, 9, 11) found that the bone age of some patients was normal or even delayed. Liang et al. (11) established that the proportion of the advanced bone age group was 32.65% (16/49) based on the difference between the bone age and the chronological age being >1 year. These studies evidenced that advanced bone age was not one of the typical characteristics in *ACAN* variant carriers. In this study, one case (1/7) had an advanced bone age, whereas the bone age of the other patients was normal; no significant acceleration in bone age was established during the rhGH treatment. It is noteworthy that Sentchordi-Montane et al. (17) even suggested that the identifications of advanced bone age and osteoarticular complications were not necessary for the diagnosis of aggrecan-associated dysplasias. For comprehensiveness considerations, advanced bone age and osteoarthritis do occur in a certain proportion of patients with short stature, caused by *ACAN* variants, which could contribute to disease diagnosis; however, they are not obligatory conditions for making the diagnosis.

It was reported that the sex ratios of *ACAN* variant carriers were roughly equal (11), while here in our study the proportions of males in 21 *ACAN* carriers was slightly higher and the seven probands were all boys. Two reasons may be related to the above-mentioned phenomenon: (1) China's national conditions caused that the society generally had higher expectations of male height, especially most families influenced by the traditional ideology of patriarchal patriarchalism and economic factors, resulting in that boys with short stature were relatively more valued and the willingness of their parents' to improve their height was more strong. Therefore, visits of boys with short stature were more active, and male bias appeared. (2) The limited sample size of our study did not reflect the real sex ratio of patients.

In terms of treatment of this disease, the height of patients has been shown to benefit from the early use of rhGH and the timely use of gonadotropin-releasing hormone analogs (GnRHa), as well as from their appropriately combined application with aromatase inhibitors in some male patients to inhibit the progression of bone age (9, 11, 12, 18–20). A previous study (11) revealed that appropriate growth-promoting treatment improved the height of patients with heterozygous *ACAN* variants; the therapeutic efficacy was not associated with the treatment plan and variant type. Scarce research has been dedicated on investigations on the adult height of *ACAN* variant carriers; reports of only a few cases have been published until now. For example, Gkourogianni et al. (12) reported that the average height (−2.5 SDS) of five adults who had received rhGH treatment was slightly greater than that (−3.0 SDS) of 65 adults who had not receive rhGH treatment. Additionally, Van der Steen et al. (18) found that two patients with *ACAN* variants treated with a combination of rhGH and GnRHa were taller by 5–8 cm in their adulthood years than their parents that had the same variants. Regarding the relationship between the efficacy and the course of treatment, Gkourogianni et al. (12) established no significant difference in the average height increase during the first, second, and third years of the rhGH treatment; the authors considered that the therapeutic efficacy was similar to that of ISS. In research (9) reviewing the response to rhGH treatment in 26 patients, 50% of the patients showed a moderate to good response in the first year of treatment (height SDS increase > 0.35). However, a poor response after 10 years of age was observed in more patients, which indicated the benefit of early treatment, especially for patients with advanced bone age. The seven patients included in this study received rhGH treatment and were then regularly followed up. Of them, patient 7 did not come at the follow-up visit. The height of the remaining six patients was significantly improved by the treatment, indicating its effectiveness. Notably, patient 3 had the longest treatment time (5.25 years); the growth velocity accelerated in the first 0.5 years, slowed down in next 2.5 years, and then accelerated again in the subsequent 0.5 years, followed by another slow-down period in the last 1.5 years. Remarkably, the average growth velocity in this patient was 7.1 cm/year, with a satisfactory overall effect. Nevertheless, the average treatment time for all patients was relatively short, and long-term follow-up was still needed to observe the height change to prove the effect of rhGH treatment. Currently, most of the research on the treatment of patients with *ACAN* variants has been largely represented by case reports, whereas the long-term treatment data has been rather limited (9, 11, 12, 18–20). Individual responses to rhGH may be difficult to judge due to different growth patterns in childhood, preadolescence, and adolescence. Therefore, long-term large-sample randomized controlled studies are needed in the future to further confirm the response to rhGH treatment for patients with *ACAN* variants.

In addition, the curative effect of rhGH could be explained from the perspective of the growth plate development. As mentioned above, children's bone growth was centered on the growth plate, with endocrine signals, nutrition, inflammatory cytokines, extracellular fluid, paracrine signals, extracellular matrix, intracellular mechanisms, and other factors jointly regulating the proliferation and differentiation of the growth plate chondrocytes (1). Aggrecan is a component of the cartilage extracellular matrix, whereas GH/IGF1 are endocrine factors that act directly on the cartilage growth plate, promoting the proliferation, hypertrophy, and maturation of chondrocytes, increasing the synthesis of the extracellular matrix protein, and enhancing the longitudinal bone growth (21). Therefore, rhGH treatment could partially compensate for the defective growth plate function and non-specifically promote the linear bone growth in short stature caused by gene variants with non-GH /IGF1 axis defects. Nevertheless, poor long-term efficacy of GH therapy alone can be observed for the repair of the growth plate function, impaired by aggrecan molecular defects. Therefore, molecular biology research is needed aimed at the restoration of normal proteoglycan aggregates, so as to provide specific treatment that improves the height of patients with *ACAN* variants.

## Conclusions

Our study found seven novel heterozygous variants in *ACAN*, expanding the spectrum of the now established pathogenic *ACAN* variants. The seven patients included had short stature, mostly normal bone age, mild skeletal abnormalities, and no joint problems; one of them was born with SGA. All cases had a family history of short stature, with or without osteoarthritis or intervertebral disc disease. The RhGH treatment applied here effectively improved the patient height, but the long-term effect of the therapy still needs further follow-up examinations and observations. Therefore, in clinical practice, prompt genetic diagnosis and drug intervention in ISS or SGA patients with persistent short stature are recommended, especially for those with a family history of multiple short stature patients, with or without osteoarthritis or intervertebral disc disease.

## Data Availability Statement

The datasets presented in this study can be found in online repositories. The names of the repository/repositories and accession number(s) can be found in the GenBank repository: ncbi.nlm.nih.gov/genbank/, accession number OM363650–OM363670.

## Ethics Statement

The studies involving human participants were reviewed and approved by the Ethics Committee of Tianjin Medical University General Hospital. Written informed consent to participate in this study was provided by the participants' legal guardian/next of kin. Written informed consent was obtained from the individual(s), and minor(s)' legal guardian/next of kin, for the publication of any potentially identifiable images or data included in this article.

## Author Contributions

LJ and GL contributed to the conception and design of the study. JS and LJ wrote the first draft of the manuscript. JZ and LN performed the statistical analysis. CM wrote sections of the manuscript. All authors contributed to the manuscript revision and read and approved its final version for submission.

## Conflict of Interest

The authors declare that the research was conducted in the absence of any commercial or financial relationships that could be construed as a potential conflict of interest.

## Publisher's Note

All claims expressed in this article are solely those of the authors and do not necessarily represent those of their affiliated organizations, or those of the publisher, the editors and the reviewers. Any product that may be evaluated in this article, or claim that may be made by its manufacturer, is not guaranteed or endorsed by the publisher.
