# Supplementary material for: Evaluation of Growth Hormone Therapy in Seven Chinese Children With Familial Short Stature Caused by Novel ACAN Variants
Source: Front Pediatr. 2022 Mar 7;10:819074. doi: 10.3389/fped.2022.819074 (PMC8940281; doi:10.3389/fped.2022.819074)
Supplement: Supplementary file 1 [file Data_Sheet_1.docx]

**SUPPLEMENTARY TABLE 1**. The detailed follow-up information of the six patients treated.

| **Patient number** | **Age of follow-up**  **/y** | **Height**  **/cm** | **Weight**  **/kg** | **Radiological Examination** | | | |  | | **Laboratory Test** | | | | | | | |  | | | **Treatment** | |
| --- | --- | --- | --- | --- | --- | --- | --- | --- | --- | --- | --- | --- | --- | --- | --- | --- | --- | --- | --- | --- | --- | --- |
|  |  |  |  | **BA**  **/y** | **Scoliosis** | **Pituitary MRI** |  | | **Blood routine** | | **Liver and kidney function** | **Blood electrolytes** | **Thyroid function** | **Fast blood glucose and insulin** | **IGF1**  **(SDS)**  **/ng**$\boldsymbol{\cdot}$**ml^-1^** | **IGFBP3**  **(SDS)**  **/ug**$\boldsymbol{\cdot}$**ml^-1^** | **25-OHD3**  **/nmol**$\boldsymbol{\cdot}$**L^-1^** | |  | **Vitamin D/IU**$\boldsymbol{\cdot}$**d^-1^** | | **rhGH**  **/IU**$\boldsymbol{\cdot}$**(kg**$\boldsymbol{\cdot}$**d)^-1^** |
| **P1** | 3 | 84 | 10.5 | 2.6 | - | N |  | | N | | N | N | N | N | 37(-0.9) | 2.81(0.8) | 27.1 | |  | 800 | | 0.15 |
|  | 3.3 | 87.3 | 11.5 | 2.9 | — | — |  | | N | | — | — | N | N | 49(-0.4) | 3.11(1.1) | 90.5 | |  | 0 | | 0.15 |
|  | 3.5 | 88.8 | 13 | — | - | — |  | | N | | N | N | N | N | 154(1.8) | 4.19(2.0) | 54.3 | |  | 400 | | 0.15 |
| **P2** | 3.1 | 83.2 | 11.5 | 3.6 | - | N |  | | N | | N | N | N | N | 56(-0.1) | 3.55(1.5) | 85.3 | |  | 0 | | 0.15 |
|  | 3.4 | 86.7 | 13 | 3.7 | — | — |  | | N | | — | — | N | N | 142(1.6) | 4.64(2.3) | 70.5 | |  | 400 | | 0.15 |
|  | 3.8 | 90.5 | 14 | — | - | — |  | | N | | N | N | N | N | 102(0.7) | 4.17(>3) | 44.9 | |  | 800 | | 0.14 |
| **P3** | 2.9 | 85.5 | 11 | 2 | - | N |  | | N | | N | N | N | N | 60(0) | 2.51(0.4) | 60.7 | |  | 400 | | 0.15 |
|  | 3.2 | 88 | 12 | — | — | — |  | | N | | — | — | N | N | 138(1.5) | 4.34(2.1) | 65.5 | |  | 400 | | 0.14 |
|  | 3.4 | 91.5 | 13 | — | - | — |  | | N | | N | N | N | N | 151(1.7) | 5.12(2.7) | 70.3 | |  | 400 | | 0.14 |
|  | 3.8 | 94.2 | 14.5 | 3.3 | — | — |  | | N | | — | — | N | N | 204(2.1) | 4.15(>3) | 83.2 | |  | 0 | | 0.13 |
|  | 4.2 | 96.6 | 14.5 | — | - | N |  | | N | | N | N | N | N | 253(2.5) | 4.23(>3) | 56.8 | |  | 400 | | 0.13 |
|  | 4.7 | 98.8 | 15 | 3.9 | — | — |  | | N | | — | — | N | N | 186(1.3) | 3.98(2.1) | 62.4 | |  | 400 | | 0.15 |
|  | 4.9 | 101 | 15.5 | — | - | — |  | | N | | N | N | N | N | 189(1.3) | 4.17(2.4) | 59.6 | |  | 400 | | 0.16 |
|  | 5.2 | 103 | 17.5 | — | — | — |  | | N | | — | — | N | N | 224(1.7) | 4.65(3.0) | 73.0 | |  | 400 | | 0.14 |
|  | 5.6 | 106.1 | 18 | 4.8 | - | N |  | | N | | N | N | N | N | 173(0.7) | 3.83(1.3) | 64.9 | |  | 400 | | 0.18 |
|  | 6.1 | 109 | 20 | — | — | — |  | | N | | — | — | N | N | 239(1.3) | 4.67(2.2) | 64.4 | |  | 400 | | 0.17 |
|  | 6.4 | 113 | 23 | — | - | — |  | | N | | N | N | N | N | 312(1.9) | 4.96(2.5) | 51.9 | |  | 400 | | 0.17 |
|  | 6.7 | 114.4 | 22.5 | 6.3 | — | — |  | | N | | — | — | N | N | 163(-0.1) | 2.83(-0.5) | 55.3 | |  | 400 | | 0.20 |
|  | 7.7 | 120.4 | 26 | 8.2 | - | N |  | | N | | N | N | N | N | 302(1.4) | 5.58(2.4) | 49.5 | |  | 800 | | 0.19 |
|  | 8.2 | 122.7 | 26.5 | — | — | — |  | | N | | — | — | N | N | 252(1.0) | 5.86(2.7) | 45.6 | |  | 800 | | 0.19 |
| **P4** | 6 | 103.1 | 17.5 | 6.8 | - | N |  | | N | | N | N | N | N | 151(0.4) | 4.54(2.1) | 40.7 | |  | 800 | | 0.15 |
|  | 6.3 | 105.8 | 18.5 | — | — | — |  | | N | | — | — | N | N | 216(1.2) | 4.83(2.4) | 58.6 | |  | 400 | | 0.16 |
|  | 6.8 | 110.9 | 20 | — | - | — |  | | N | | N | N | N | N | 303(1.0) | 7.18(2.3) | 41 | |  | 800 | | 0.17 |
|  | 7.3 | 116 | 23 | 8.2 | — | — |  | | N | | — | — | N | N | 354(1.2) | 6.80(2.1) | 47.5 | |  | 800 | | 0.17 |
|  | 7.7 | 120 | 26 | 8.5 | - | N |  | | N | | N | N | N | N | 372(2.0) | 6.39(>3) | 56.0 | |  | 400 | | 0.17 |
| **P5** | 9.4 | 125 | 35.7 | 10 | - | N |  | | N | | N | N | N | N | 205(0.1) | 5.28(1.6) | 33.1 | |  | 800 | | 0.15 |
|  | 9.7 | 126.8 | 37.9 | — | — | — |  | | N | | — | — | N | N | 123(-0.1) | 4.78(1.3) | 48.7 | |  | 800 | | 0.16 |
|  | 10 | 130 | 40 | 10.7 | - | — |  | | N | | N | N | N | N | 346(1.5) | 6.94(2.6) | 45.2 | |  | 800 | | 0.16 |
|  | 10.3 | 133.8 | 39.4 | — | — | — |  | | N | | — | — | N | N | 332(1.4) | 5.87(2.1) | 50.6 | |  | 400 | | 0.15 |
|  | 10.6 | 136 | 41.5 | — | - | N |  | | N | | N | N | N | N | 239(0.7) | 5.15(1.6) | 55.3 | |  | 400 | | 0.15 |
|  | 11.1 | 140 | 45 | 12.0 | — | — |  | | N | | — | — | N | N | 315(1.1) | 5.82(2.1) | 60.4 | |  | 400 | | 0.15 |
|  | 11.4 | 143.8 | 48 | — | - | — |  | | N | | N | N | N | N | 367(1.2) | 6.29(2.4) | 63.5 | |  | 400 | | 0.14 |
|  | 11.6 | 145 | 54 | 12.5 | — | — |  | | N | | — | — | N | N | 352(0.7) | 5.76(1.7) | 64.8 | |  | 400 | | 0.15 |
|  | 11.9 | 147.1 | 54.6 | — | - | N |  | | N | | N | N | N | N | 421(1.0) | 6.91(2.4) | 70.9 | |  | 400 | | 0.15 |
|  | 12.2 | 148.7 | 57 | 13 | — | — |  | | N | | — | — | N | N | 364(0.8) | 6.25(2.0) | 73.7 | |  | 400 | | 0.16 |
| **P6** | 3.4 | 89 | 13.5 | 3.8 | - | N |  | | N | | N | N | N | N | 91(0.8) | 3.44(1.4) | 112.6 | |  | 0 | | 0.15 |
|  | 3.7 | 91.3 | 14.5 | — | — | — |  | | N | | — | — | N | N | 138(1.3) | 3.52(2.1) | 84.5 | |  | 0 | | 0.15 |
| Abbreviations: P, patient; y, years old; BA, bone age; MRI, magnetic resonance imaging; SDS, standard deviation score; IGF1, insulin-like growth factor 1; IGFBP3, insulin-like growth factor-binding protein 3; 25-OHD3, 25-hydroxy vitamin D3; rhGH, recombinant human growth hormone; N, normal; ‘-’ indicates “no”. | | | | | | | | | | | | | | | | | | | | | | |

| **Patient number** | **P1** | **P2** | **P3** | **P4** | **P5** | **P6** | **P7** |
| --- | --- | --- | --- | --- | --- | --- | --- |
| **Nucleotide Change** | c.1051+2T > A | c.313T > C | c.2660C > G | c.2153C > A | c.7243delG | c.2911G > T | c.758-7T > C |
| **Protein change** | — | p.S105P | p.S887X | p.T718K | p.D2415Tfs*4 | p.G971X | — |
| **Exon/Intron** | Intron 6 | Exon 3 | Exon 12 | Exon 11 | Exon 16 | Exon 12 | Intron 5 |
| **Domain** | G1 | G1 | CS1 | KS | CLD | CS1 | G1 |
| **Variant Type** | Splicing | Missense | Nonsense | Missense | Frameshift | Nonsense | splicing |
| **Inherited** | Maternal | Maternal | Maternal | Paternal | Maternal | Paternal | Paternal |
| **ACMG** | Likely pathogenic | Uncertain | Likely pathogenic | Uncertain | Likely pathogenic | Likely pathogenic | Uncertain |
| **REVEL** | NA | Probably damaging | NA | Benign | NA | NA | NA |
| **SIFT** | NA | Damaging | NA | Damaging | NA | NA | NA |
| **PolyPhen_2** | NA | Damaging | NA | Benign | NA | NA | NA |
| **Mutation Taster** | Disease  causing | Disease causing | Disease causing | Polymorphism | Disease causing | Disease causing | NA |
| **GERP+** | NA | Conserved | NA | Non-conserved | NA | NA | NA |
| Abbreviations: P, patient; NA, not available; G1, globular domain 1; KS, keratan sulfate; CS1, chondroitin sulfate 1; CLD, C-type lectin domain; ACMG, American College of Medical Genetics and Genomics. | | | | | | | |

**SUPPLEMENTARY TABLE 2.** Genetic information of the seven patients included in the investigation.
